# Draft Genome Sequence of the Putrescine-Producing Strain *Lactococcus lactis* subsp. *lactis* 1AA59

**DOI:** 10.1128/genomeA.00669-15

**Published:** 2015-06-18

**Authors:** Victor Ladero, Beatriz del Rio, Daniel M. Linares, María Fernandez, Baltasar Mayo, M. Cruz Martín, Miguel A. Alvarez

**Affiliations:** Instituto de Productos Lácteos de Asturias, IPLA-CSIC, Paseo Río Linares, Asturias, Spain

## Abstract

We report here the 2,576,542-bp genome annotated draft assembly sequence of *Lactococcus lactis* subsp. *lactis* 1AA59. This strain—isolated from a traditional cheese—produces putrescine, one of the most frequently biogenic amines found in dairy products.

## GENOME ANNOUNCEMENT

*Lactococcus lactis* has been largely used as a main component of dairy starter cultures for the manufacture of fermented dairy products (1). In addition, it is the model lactic acid bacterium (LAB) for research on metabolism, physiology, and genetics. *L. lactis* comprises two subspecies (subsp. *lactis* and subsp. *cremoris*) (1), and both are used in the production of many types of cheese because of their capacity to produce lactate as well as taste and aroma compounds. Nevertheless, some strains of the two subspecies can produce the biogenic amine putrescine (2, 3).

Biogenic amines (BAs) are toxic compounds that can accumulate in foods and beverages due to the metabolic activity of certain microorganisms. The ingestion of foods with a high BA concentration can cause intoxication symptoms (4). Consequently, there is general consensus to reduce their presence in foods (5). Putrescine is one of the most frequent and abundant BAs in cheese, where it is mainly synthesized by the agmatine deiminase (AGDI) pathway (2). Different evolution of the AGDI pathway (3) as well as different regulatory responses of the pathway to lactose have been observed among the two *L. lactis* subspecies (6). We have previously reported the genome sequence of *L. lactis* subsp. *cremoris* CECT8666 (7) in which putrescine production is regulated by carbon catabolic repression via glucose but not lactose (6). We here report the draft genome of *L. lactis* subsp. *lactis* 1AA59—a strain isolated from a traditional artisanal cheese (8)—in which putrescine biosynthesis is repressed by glucose and lactose (6).

A 0.5-kbp genomic library was constructed and subjected to 90-bp paired-end sequencing (providing approximately 25-fold coverage) using a HiSeq 1000 system sequencer (Illumina) (performed at the Beijing Genomics Institute [China]). Quality-filtered reads were assembled using Velvet software (http://www.ebi.ac.uk/~zerbino/velvet/), resulting in 218 contigs ranging from 450 to 144,836 bp. The total sequence length is 2,576,542 bp, and the GC content is 34.9%, which is within the range of other *L. lactis* genomes (34.86 to 35.88%). Annotation was performed using the Prokaryotic Genomes Annotation Pipeline (PGAAP) application on the NCBI server (http://www.ncbi.nlm.nih.gov/genome/annotation_prok/) and improved using the results obtained in BLAST analysis (http://blast.ncbi.nlm.nih.gov). The genome contained 2,611 predicted coding sequences. Predicted copies of the 16S, 23S, and 5S rRNA genes were found, as well as 32 genes for tRNAs.

The AGDI gene cluster involved in putrescine production was identified (*aguR*, N489_06595; *aguB,* N489_06595; *aguD,* N489_06595; a*guA*, N489_06595; and *aguC,* N489_06595). Sequence comparison with the genome of the putrescine-producing *L. lactis* subsp. *cremoris* strain CECT8666 revealed the presence of 404 genes present in 1AA59 but not in CECT8666 and 482 genes present in CECT8666 but not in 1AA59. These genes include several putative transcriptional regulators and sugar metabolism-related genes.

An in-depth sequence analysis of the *L. lactis* subsp. *lactis* 1AA59 genome combined with transcriptional studies will help to identify the regulatory pathways involved in the production of putrescine and would help to provide clues toward the reduction of BA presence in dairy products.

### Nucleotide sequence accession numbers.

The results of this whole-genome shotgun project were deposited in the DDBJ/EMBL/GenBank database under the accession number AZQT00000000 (BioProject PRJNA213570). The version of the genome described here is version AZQT00000000.1.
